# The Component Separation Index: A Standardized Biometric Identity in Abdominal Wall Reconstruction

**Published:** 2012-03-22

**Authors:** Michael R. Christy, John Apostolides, Eduardo D. Rodriguez, Paul N. Manson, David Gens, Thomas Scalea

**Affiliations:** ^a^Division of Plastic and Reconstructive Surgery, R Adams Cowley Shock Trauma Center The University of Maryland Medical Center, Baltimore, MD; ^b^The John Hopkins Hospital Department of Plastic and Reconstructive Surgery, Baltimore, MD

## Abstract

**Objective:** Reconstruction of traumatic ventral hernias often requires additional techniques to the abdominal wall component separation, such as the use of interpositional reconstruction with an acellular dermal matrix or other mesh to bridge the defect. **Methods:** We have developed a new value termed the “Component Separation Index” to evaluate ventral hernia defects. Choosing a fixed point on a preoperative axial computed tomographic scan (aorta) and the medial leading edges of the rectus abdominus muscles, we determined the angle of diastasis of the hernia. This angle is divided by 360° giving a relative value of the transverse defect size as compared to the estimated circular body habitus for that specific patient. A retrospective review of 36 cases of ventral hernia repairs was performed. The Component Separation Index was calculated from the preoperative computed tomographic scans obtained before repair. Group 1 (n = 18) required component separation for closure. Group 2 (n = 18) required component separation and placement of interpositional mesh to span the hernia defect. **Results:** The Component Separation Index values were then compared using the student *t* test for each group. The mean Component Separation Index for group 1 was 0.11 with standard deviation of 0.06. The mean Component Separation Index for group 2 was 0.21 with standard deviation of 0.04 (*P* < .0001). As this value approaches 0.21, the likelihood of an interpositional repair in addition to component separation becomes much greater. **Conclusions:** While there is no substitute for clinical acumen when evaluating these defects, objective measurements can provide a valuable additional tool for the surgeon facing these challenging cases.

The vertical midline incision via the linea alba remains the standard technique for abdominal surgery. Incidence of herniation after midline laparotomy varies in reports from 2% to 11%.^1^^,^^2^ Early reports of reconstruction of these deficits with *direct closure* techniques yielded disappointing results, with failure rates of up to 50%.^3^ To address these results several authors published techniques utilizing the multilayered *myofascial envelope* of the abdominal wall to alleviate the tension in abdominal wall closure.^4^^-^11^,^^13^^-^42 The “Component Separation” technique for repair of ventral hernias was first described by Ramirez et al in 1990.^4^ This was a formal description of a selective release of abdominal fascia to assist with closure of ventral hernias. The technique includes separation of the rectus abdominus muscle from the external oblique muscle with dissection in the avascular plane between the external oblique and the internal oblique muscles. Since that time there have been several developments regarding the technique, and several variations of the procedure have been described.^5^^-^8 Several large reviews of the literature also detail methods of component separation and comparisons of outcomes with various types of mesh.^18^^,^^19^^,^^21^^,^^23^^,^^25^^-^30 The benefits of this procedure focus on its use of innervated, vascularized, autologous tissue for the reconstruction of the abdominal wall. In addition, beyond providing for a reduction in tension, the use of these innervated, myofascial flaps helps to recreate the dynamic nature of the native abdominal wall. These and other reconstructive strategies have contributed to a body of literature citing a recurrence rate now averaging 10% to 15%.^5^^-^7 Preoperative assessment of the ventral hernia patients reveals significant loss of domain, poor quality of the surrounding skin and soft tissue envelope, and a significant rectus muscle diastasis. One of the challenges when planning an abdominal wall reconstruction is predicting the need for interpositional mesh repair versus performing a component separation to achieve midline closure. In an attempt to address this problem, we are proposing the Component Separation Index (CSI), a calculated value based on the preoperative computed tomographic (CT) scans of these patients. The concept of an index value is seen throughout the medical literature, examples of which include the ankle-brachial index used in vascular surgery and the body mass index used in bariatric surgery. These values arose out of the need to quantify biometric data and provide a useful objective value for preoperative evaluation, comparison, or as predictors of outcomes. Regarding repair of large ventral hernias, rather than relying only on the distance between the edges of the hernia or the total square centimeters of the defect, the CSI utilizes a value based on a preoperative CT scan of the abdomen with the *angle of diastasis* of the rectus musculature with the vertex based at the aorta. This is then placed as a comparator over 360°. The value of the angle of diastasis takes into account not only the transverse dimension of the hernia but also the AP dimension of the patients' unique body habitus providing an added dimension of the patients' biometric identity. (Figs 1 and 2)

## METHODS

### Patient enrollment

A retrospective review of all patients undergoing ventral hernia repair were included in the study in accordance with the patient population of the senior authors (M.C., E.R). Approximately 80% of the ventral hernia patients had a CT scan performed preoperatively and had identifiable medial edges of the rectus abdominus muscles allowing for a calculation of angle of diastasis. We reviewed operative reports of all patients included in the study. Two groups were created on the basis of type of ventral hernia repair. Group 1 consisted of patients that required component separation alone without the use of interpositional mesh (n = 18). Group 2 consisted of all patients requiring interpositional mesh in addition to component separation (n = 18). Approval of institutional review board was obtained for evaluation of CT scans and review of operative reports in accordance with the guidelines set forth by the University of Maryland Medical Center/R Adams Cowley Shock Trauma Center institutional review board.

Group 1 (n = 18) required component separation for closure without the need for interpositional repair. The technique used for component separation included a bilateral longitudinal release of the fascia between the rectus abdominus muscle and the external oblique muscle.

Group 2 (n = 18) required component separation and placement of interpositional mesh to span the residual hernia defect. The technique used for component separation included a bilateral longitudinal release of the fascia between the rectus abdominus muscle and the external oblique muscle. However, in this group there was inadequate laxity using the component separation technique alone to permit midline reapproximation of the rectus abdominus muscles and reconstruction of the abdominal wall under reasonable physiologic tension. In all cases, the interpositional repair consisted of a bioprosthesis (dermal matrix) with or without an additional overlay repair with prolene mesh.

#### The angle of diastasis

**Figure 1:**
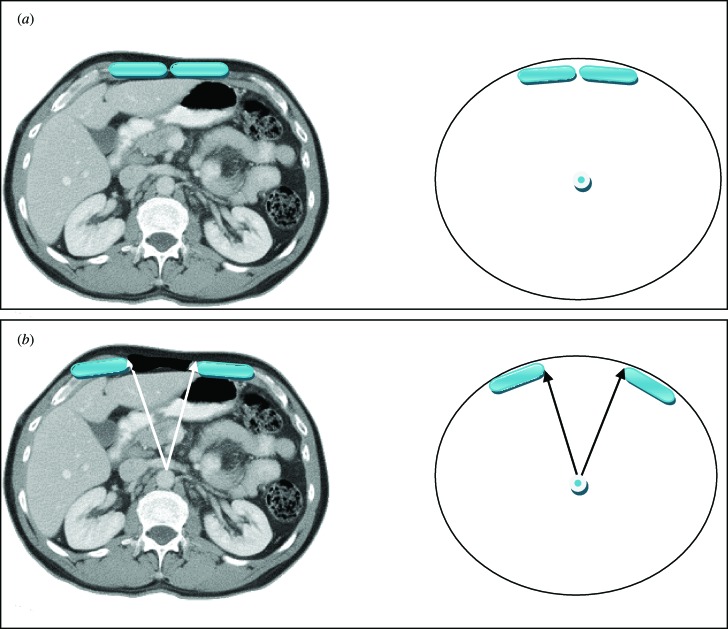
(*a*) Axial CT scan of the normal anatomic position of the rectus abdominus muscles showing minimal midline separation. (*b*) Axial CT scan of a patient with a rectus diastasis demonstrating an increased distance between the rectus abdominus muscles.

**Figure 2:**
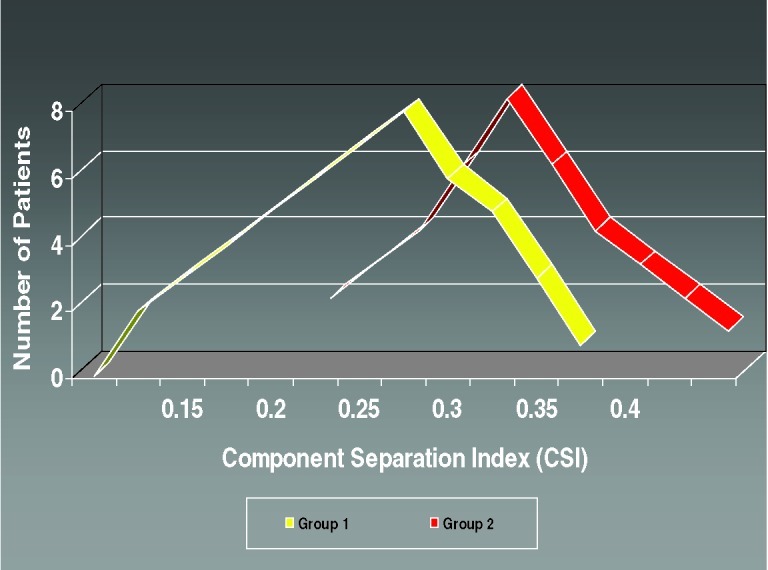
Group 1 (n = 18) required component separation for closure without the need for interpositional repair. The technique used for component separation included a bilateral longitudinal release of the fascia between the rectus abdominus muscle and the external oblique muscle. Group 2 (n = 18) required component separation and placement of interpositional mesh to span the residual hernia defect. The technique used for component separation included a bilateral longitudinal release of the fascia between the rectus abdominus muscle and the external oblique muscle. However, in this group there was inadequate laxity using the component separation technique alone to permit midline reapproximation of the rectus abdominus muscles.

This angle was measured from the preoperative CT scans obtained for each patient included in the study. The vertex of the angle was taken as the position of the aorta at the axial CT image where the maximal transverse dimension of the hernia defect was identified. The arms of the angle were then taken from this point to the medial edges of the rectus abdominus muscles (Figs 1 and 2).

#### Component Separation Index

The index value was then calculated with the angle of diastasis (AD) as the numerator and 360 as the denominator.

CSI = AD/360

## RESULTS

**Figure 3:**
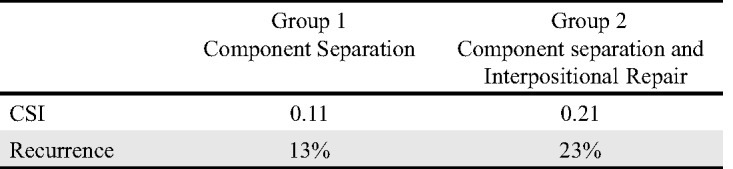
Group 1 and group 2 CSI values and ventral hernia recurrence rate.

In our retrospective review, 18 patients were identified who underwent only the component separation technique for abdominal hernia repair without the use of interpositional mesh. A separate group of 18 patients was identified who underwent component separation and interpositional mesh reconstruction. The charts were reviewed and patient data were studied. The age of our study population ranged from 17 to 85 years (mean age, 50 years), in which 51% were female and 49% were male. The average follow-up time was 36 months (range, 12-96 months). The average defect size was large, equaling 155 cm^2^. The mean CSI values for group 1 and group 2 were then compared using the student *t* test. Using the preoperative abdominal CT scans, the angle of diastasis was measured using the software included in the IMPAX system. The CSI was then calculated as described previously, and the groups were compared. The 18 patients identified for group 1 had a mean CSI of 0.11 with a standard deviation of 0.06. Group 2, also 18 patients, had a mean CSI of 0.21 with a standard deviation of 0.04 (*P* < .0001). For all repair techniques, the overall recurrence rate was 19%. Recurrence was directly related to the extent of repair required. Group 1 patients experienced a recurrence rate of 13%; in group 2, the recurrence rate was 23%. Postoperative complications varied but most frequently they involved wound infection. The frequency of wound infection was directly related to the extent of repair required (Figure 3).

## DISCUSSION

Large traumatic ventral hernias continue to challenge the reconstructive plastic surgeon. There have been no published data or reports regarding an objective clinical score system or predictive value for the management of abdominal wall reconstruction. Current literature has focused on basic size of defect in the horizontal and/or vertical direction or on area of defect combined with some technical variations in repair. This allows for a single, independent analysis of a single patient and their defect. These studies lack a standardized metric to permit a truly objective analysis. Throughout the medical and the plastic surgery literature, anthropomorphic measurements have been used to assess outcomes and predict treatment algorithms and complications. This concept was recently demonstrated by Lahiri et al^12^ regarding abdominoplasty and reduction mammaplasty. In addition, the body mass index has been shown to have a predictive value for surgical complications. In reviewing our data for abdominal wall reconstruction for large traumatic ventral hernias, we sought to create a standardized value to allow for true comparison and analysis of these reconstructive dilemmas.

Scar tissue that develops between the layers of the myofascial envelope of the abdominal wall will likely change the differential distances one can achieve with an abdominal component release. The CSI is limited in that it will not account for this variable. Our goals were to review the large ventral hernias seen in our trauma center and find an objective method to predict whether we would need to perform only a component separation versus a component separation with use of interpositional mesh (consisting of an acellular dermal matrix with or without a composite of prosthetic mesh). There are limitations with the CSI value. Certainly with these cases, there may be changes in the original surgical plan as scarring of the rectus muscles and between the rectus fascia and the overlying soft tissue envelope can be extensive. This may limit advancement of the myofascial components toward the midline. Loss of domain also plays a role in the algorithm. Increase of intraoperative peak airway pressure may alter the original plan of component separation to the addition of interpositional repair. The CSI represents a percentage loss of abdominal domain specific to each patient's body habitus. The CSI takes into account the size of the hernia defect relative to the remaining tissue present in the abdominal wall. The calculated value serves as an accurate biometric assessment of the abdominal wall defect rather than an absolute value of the distance between leading edge of rectus muscle and the area of the defect. By creating a value that correlates defect size to each patient's unique biometric profile, we create a standardized value that allows for true comparison. Postoperative recurrence rates were also evaluated in our patient population. Not surprisingly, we found that there is also an association with the CSI angle of diastasis and the rate of hernia recurrence.

With the efficiency of today's CT imaging, preoperative CT scans are regularly obtained in preparation of the ventral hernia patient mostly for information for the general surgeon regarding the status of the abdominal viscera and adhesions. Information on the status of the myofascial components of the abdominal wall are therefore readily available most of the time for the plastic surgeon planning on a component separation. In analysis of axial slices of abdominal CT scans, we can visualize the rectus abdominus diastasis and locate our lead points for the component separation. Therefore, we can take a standard midpoint, aorta, and translate the transverse defect size into a percentage of abdominal domain loss. A fixed defect size will represent a different severity of abdominal domain loss based on patient body habitus. The CSI assigns a conceptual numeric value addressing the 3-dimensional nature of the ventral hernia.

## CONCLUSIONS

Abdominal wall reconstruction in the ventral hernia patient represents a constant challenge to the general surgeon and plastic and reconstructive surgeon. Current studies lack a predictive value or objective 3-dimensional measurement to assist in the treatment algorithm or to assess and analyze surgical outcomes. On the basis of our current data, we believe the CSI, much like other commonly used anthropomorphic measurements, can assist with decisions for reconstructive options preoperatively. Future studies will focus on the CSI as a value to assist in postoperative outcome assessment of these complex clinical problems. In doing so, we hope to create a predictive value which will assist surgeons in their approach to the surgical treatment of ventral hernias.
